# Circulating and disseminated tumor cells from breast cancer patient-derived xenograft-bearing mice as a novel model to study metastasis

**DOI:** 10.1186/s13058-014-0508-5

**Published:** 2015-01-09

**Authors:** Mario Giuliano, Sabrina Herrera, Pavel Christiny, Chad Shaw, Chad J Creighton, Tamika Mitchell, Raksha Bhat, Xiaomei Zhang, Sufeng Mao, Lacey E Dobrolecki, Ahmed Al-rawi, Fengju Chen, Bianca M Veneziani, Xiang H-F Zhang, Susan G Hilsenbeck, Alejandro Contreras, Carolina Gutierrez, Rinath M Jeselsohn, Mothaffar F Rimawi, C Kent Osborne, Michael T Lewis, Rachel Schiff, Meghana V Trivedi

**Affiliations:** Lester and Sue Smith Breast Center, Baylor College of Medicine, Houston, TX USA; Department of Clinical Medicine and Surgery, University Federico II, Naples, Italy; Department of Pharmacy Practice and Translational Research, University of Houston, Houston, TX USA; Dan L. Duncan Cancer Center, Baylor College of Medicine, Houston, TX USA; Department of Molecular and Human Genetics, Baylor College of Medicine, Houston, TX USA; Department of Medicine, Baylor College of Medicine, Houston, TX USA; Department of Molecular Medicine and Medical Biotechnology, University Federico II, Naples, Italy; Department of Molecular and Cellular Biology, Baylor College of Medicine, Houston, TX USA; Department of Pathology, Baylor College of Medicine, Houston, TX USA; Dana-Farber Cancer Institute, Harvard Medical School, Boston, MA USA; Department of Radiology, Baylor College of Medicine, Houston, TX USA; Department of Pharmacological and Pharmaceutical Sciences, University of Houston, Houston, TX USA

## Abstract

**Introduction:**

Real-time monitoring of biologic changes in tumors may be possible by investigating the transitional cells such as circulating tumor cells (CTCs) and disseminated tumor cells in bone marrow (BM-DTCs). However, the small numbers of CTCs and the limited access to bone marrow aspirates in cancer patients pose major hurdles. The goal of this study was to determine whether breast cancer (BC) patient-derived xenograft (PDX) mice could provide a constant and renewable source of CTCs and BM-DTCs, thereby representing a unique system for the study of metastatic processes.

**Methods:**

CTCs and BM-DTCs, isolated from BC PDX-bearing mice, were identified by immunostaining for human pan-cytokeratin and nuclear counterstaining of red blood cell-lysed blood and bone marrow fractions, respectively. The rate of lung metastases (LM) was previously reported in these lines. Associations between the presence of CTCs, BM-DTCs, and LM were assessed by the Fisher’s Exact and Cochran-Mantel-Haenszel tests. Two separate genetic signatures associated with the presence of CTC clusters and with lung metastatic potential were computed by using the expression arrays of primary tumors from different PDX lines and subsequently overlapped to identify common genes.

**Results:**

In total, 18 BC PDX lines were evaluated. CTCs and BM-DTCs, present as either single cells or clusters, were detected in 83% (15 of 18) and 62.5% (10 to16) of the lines, respectively. A positive association was noted between the presence of CTCs and BM-DTCs within the same mice. LM was previously found in 9 of 18 (50%) lines, of which all nine had detectable CTCs. The presence of LM was strongly associated with the detection of CTC clusters but not with individual cells or detection of BM-DTCs. Overlapping of the two genetic signatures of the primary PDX tumors associated with the presence of CTC clusters and with lung metastatic potential identified four genes (*HLA-DP1A*, *GJA1*, *PEG3*, and *XIST*). This four-gene profile predicted distant metastases-free survival in publicly available datasets of early BC patients.

**Conclusion:**

This study suggests that CTCs and BM-DTCs detected in BC PDX-bearing mice may represent a valuable and unique preclinical model for investigating the role of these rare cells in tumor metastases.

**Electronic supplementary material:**

The online version of this article (doi:10.1186/s13058-014-0508-5) contains supplementary material, which is available to authorized users.

## Introduction

Circulating tumor cells (CTCs) are cancer cells originating from either a primary or metastatic tumor and circulating freely in the peripheral blood [1]. It has been proposed that the spread of a primary tumor through the bloodstream as CTCs is a critical step in tumor metastasis [2,3]. In breast cancer (BC), CTCs can be detected in patients at early stages or late stages of disease with overt metastases [4-6]. Many studies have shown that the detection of CTCs may help to predict the outcome in patients with different types of cancers. In particular, the enumeration of CTCs before starting systemic treatment is associated with clinical outcome in both metastatic and non-metastatic BC patients [4,6,7]. Furthermore, CTC count evaluated at different time points during systemic treatment is a reliable surrogate marker of treatment response [8-12]. Preliminary studies have suggested that selecting therapies based on molecular characteristics of CTCs may improve treatment outcomes in patients [13-15]. Because CTCs are found in circulation as a collectable fraction that is representative of the tumor, they may provide an ideal model to study the biology of the tumor at various intervals before and during treatment [16].

Interestingly, the presence of CTCs has been found to correlate with the presence of disseminated tumor cells in the bone marrow (BM-DTCs) in BC patients [17,18]. Similar to CTCs, BM-DTCs play a crucial role in the metastatic cascade as the earliest detectable form of micrometastatic disease and potential precursors of overt metastases [19]. Notably, several studies have shown that persistence of BM-DTCs after therapy predicts a higher risk of relapse in BC patients [20,21]. Therefore, BM-DTCs represent an additional tool for studying the metastatic process in its initial stage.

Despite the evidence to support the roles of CTCs and BM-DTCs in studying tumor biology and predicting treatment response, routine clinical and preclinical use of these cells is challenging because of multiple factors. First, CTCs are present in small numbers in only 10% to 50% of BC patients [22-24]. Therefore, they cannot be isolated in sufficient numbers from a small volume of blood from most patients. Similarly, a longitudinal study of BM-DTCs is impractical because of limited access to bone marrow aspirates and the small number of DTCs that can be enriched from aspirates of standard volume. In addition, commonly used methods to detect CTCs use antibodies against epithelial cell markers and exclude identification of tumor cells with mesenchymal properties. This is especially problematic, as the cells that have undergone epithelial-to-mesenchymal transition (EMT) may play an essential role in the metastatic process [25].

To address these major challenges, we aimed to determine whether BC patient-derived xenograft (PDX)-bearing mice could provide a constant and renewable source of CTCs and BM-DTCs as a unique system to study the molecular changes responsible for tumor progression and metastases. Here, we report the detection of human CTCs and BM-DTCs in various BC PDX mice models [26]. To identify the PDX lines with high numbers of CTCs and BM-DTCs, we screened a total of 18 lines representing different molecular subclasses of BC. Further, we evaluated the association of CTC detection with the presence of BM-DTCs and with the lung metastatic potential of these PDX lines. Finally, we determined the predictive value of a genetic profile computed from the primary tumors of various PDX lines that was associated with the presence of CTC clusters and lung metastatic potential.

## Methods

### BC PDX mouse models

BC PDX mouse models were established in Dr. Michael Lewis’ laboratory at the Lester and Sue Smith Breast Center in Baylor College of Medicine. Methods used to establish these PDX models have been recently reported [26]. Mice transplanted with tumors from passages 2 through 11 were used for our studies. Animal care for the mice bearing the BC PDX tumors, as well as age- and gender-matched control mice, was in accordance with the NIH Guide for the Care and Use of Experimental Animals with approval from the Baylor College of Medicine Institutional Animal Care and Use Committee.

In brief, mammary fat pad epithelium was surgically cleared from 3- to 4-week-old SCID/Beige female mice. Subsequently, fresh breast tumor fragments collected directly from patients were orthotopically transplanted into the cleared mammary fat pads. When tumor size reached 1,000 mm^3^, mice were killed, and tumors were re-transplanted into additional mice up to 11 passages. Importantly, the primary and serially passaged PDXs have shown genomic, proteomic, phenotypic, and histologic consistency with the tumor of origin, and they are also genetically and proteomically stable across multiple transplant generations [26].

### Detection of CTCs, BM-DTCs, and lung metastases

After anesthesia, peripheral blood (500 to 700 μl) was collected from the *vena cava inferior* of each animal, which was then killed by cervical dislocation. Tibias, femurs, and hip-bones were collected, and bone marrow was flushed from the bones by using phosphate-buffered saline (PBS) supplemented with 2 m*M* ethylenediaminetetraacetic acid (EDTA). Whole blood was processed within 1 hour of collection to lyse red blood cells (RBCs) by incubation with ammonium chloride (StemCell Technologies, Vancouver, BC, Canada) per manufacturer’s protocol.

RBC-depleted cells and bone marrow cells were then washed twice with PBS at room temperature, pelleted, and fixed with 10% neutral buffered formalin for 3 hours at room temperature. Cell pellets were embedded in paraffin and cut in consecutive sections of 5-μm thickness. Five consecutive sections of every 15 sections were stained by using immunohistochemistry (IHC) with anti-human pan-cytokeratin (clone AE1/AE3 against cytokeratins 1–8, 10, 13–16, and 19; Source: Dako, Carpinteria, CA, USA) and nuclear counterstain (hematoxylin). CTCs and BM-DTCs were identified as cytoplasmic human pan-cytokeratin-positive and nuclear counterstain-positive cells. A CTC or BM-DTC cluster was defined as a group of two or more CTCs or BM-DTCs, respectively. At least two PDX-bearing mice were tested per line (range, 2 to 7), and in total, five age-matched non-tumor-bearing female mice were used as controls. Lung metastases (LMs) were identified by IHC in these PDX lines and were reported in the previous study [26].

### Genetic signature of primary tumors associated with CTC clusters and LM

The following nine of 18 screened lines had published Affymetrix gene expression data (GEO:GSE46106), and at least three mice screened for CTCs (BCM-3107, BCM-3204, BCM-3561, BCM-3613, BCM-3887, BCM-3963, BCM-4272, BCM-4664, BCM-4888) [26]. Of these nine lines, CTC clusters were present in three lines and LM were detected in six lines (Table 1). To identify differential genetic signatures corresponding to CTC clusters and to LM, analysis of variance followed by t-tests were performed by applying linear modeling to our microarray experiments by using the Linear Models for Microarray Data (LIMMA) method, and subsequent manual curation was used, as described previously [27]. A genetic profile overlapping these two genetic signatures was derived and interrogated for prediction of distant metastasis-free survival in publicly available datasets [28].

**Table 1:** **Identification of CTCs and BM-DTCs in BC PDX lines and previously reported presence of LM**

**PDX line**	**CTC detection rate (%)**	**Number of CTCs per 20 K nucleated cells (≈20 μl of blood)**	**BM-DTC detection rate (%)**	**Number of BM-DTCs per 2 × 10** ^**6**^ **BM cells**	**LM rate** [26] **(%)**
BCM-3107*	1/4 (25)	7	1/2 (50)	1	0
BCM-3143	0/2 (0)	0	0/2 (0)	0	0
BCM-3204*	2/4 (50)^Cl^	3-43	0/2 (0)	0	29
BCM-3561*	2/4 (50)	1	ND	ND	0
BCM-3613*	1/3 (33)	7	0/2 (0)	0	24
BCM-3807	0/4 (0)	0	1/2 (50)	29	0
BCM-3887*	3/4 (75)^Cl^	3-92	1/5 (20)	<1	14
BCM-3963*	1/6 (17)	7	0/4 (0)	0	14
BCM-4195	0/2 (0)	0	0/2 (0)	0	0
BCM-4272*	3/3 (100)	<1-25	4/6 (67)^Cl^	<1-132	29
BCM-4664*	1/3 (33)	2	ND	ND	0
BCM-4888*	5/5 (100)^Cl^	3-28	0/4 (0)	0	67
BCM-5097	3/6 (50)^Cl^	10-91	3/5 (60)	12-30	36
BCM-5156	3/5 (60)	1-28	1/2 (50)	2	0
BCM-5438	2/2 (100)	2-3	2/2 (100)	4-67	0
BCM-5471	3/6 (50)^Cl^	1-10	2/4 (50)^Cl^	<1-6	33
BCM-5998	5/7 (71)	<1-27	6/8 (75)^Cl^	1-40	0
BCM-6257	3/3 (100)^Cl^	3-4	2/3 (67)^Cl^	22-39	6

BC*,* breast cancer; BM*,* bone marrow; BM-DTCs*,* disseminated tumor cells in bone marrow; CTC,s circulating tumor cells*,* LM, lung metastasis*,* ND, not determined*,* PDX, patient-derived xenograft. ^Cl^ represents presence of CTCs and BM-DTCs in clusters. * indicates the lines with published Affymetrix data.

### Statistics

The number of CTCs was reported per 20,000 nucleated cells (≈20 μl of blood). This was based on our initial testing of >20 representative mice from nine PDX models showing that the average number of RBC-depleted nucleated cells was 20,000 per 20 μl of blood collected. The DTC count was reported as number of DTCs per 2 million bone marrow cells. In addition, the CTC and BM-DTC detection rates were calculated per each PDX line as the ratio of the number of mice with one or more CTCs and DTCs, respectively, and the number of mice tested. Lung metastatic rate was previously reported as the ratio of the mice affected by metastases and the number of mice tested per each line [26]. The association between the presence of CTCs and BM-DTCs was evaluated within the same mice by using Fisher’s Exact test and Cochran-Mantel-Haenszel test to adjust for different PDX lines. The data are presented in a collapsed 2 × 2 contingency table without different strata representing different PDX lines. The association between the ability to produce CTCs or CTC cluster occurrence and the presence of LM was assessed within each line because the latter was evaluated in a previous study [26]. These associations were assessed by Fisher’s Exact test, and data were represented in 2 × 2 contingency table. To derive genetic signatures associated with CTC clusters and LM, genes that were differentially expressed and had some statistical evidence of being repeatable were considered; this signature was determined by using a linear contrast *t* test q–value (also called false discovery rate (FDR)) <0.25 *or* at least 16-fold average difference in expression between LM-positive and LM-negative PDX lines, which corresponds to a log2 contrast between LM-positive and LM-negative scoring greater than 4 in absolute value. To determine the prognostic value of the four-gene profile, gene-expression data of human BC [28] were each scored, taking the sum of the two “upregulated” genes minus the sum of the two “downregulated” genes (by using z-normalized expression values). All *P* values reported were two-sided, unless otherwise specified.

## Results

### BC PDX line characteristics

We used 18 PDX-bearing mouse models, which have been described in detail previously [26]. Sixteen of these lines (89%) were established by using primary breast tumor fragments obtained from patients without metastases. The other two lines (BCM-3561 and BCM-3613) were developed by transplanting tumor cells isolated from ascites and pleural fluid collected from two patients with metastatic disease, respectively. Among the lines we screened, one line (BCM-5097) was estrogen receptor (ER)-positive, progesterone receptor (PR)-positive, but human epidermal growth factor receptor 2 (HER2) negative; three lines were ER-negative and PR-negative, but HER2-positive (BCM-3143, BCM-3613, BCM-3963); one line was positive for ER, PR, and HER2 (BCM-4888); the remaining 13 lines (72%) were triple (ER, PR, HER2)-negative. All the tumor transplants were positive for either CK19 or CK5/6 or both, as previously reported [26].

### Detection of CTCs, BM-DTCs, and LM in BC PDX lines

All five age-matched control female mice were negative for both CTCs and BM-DTCs. Individual CTC and BM-DTC detection data in all tested mice are reported in Additional file 1: Table S1. Of 81 mice, only eight did not have adequate sample for CTC analysis and are labeled as NA (not available). The DTC analysis was initiated later when we already had collected CTC data for >20 mice. Therefore, DTC data were not available for a total of 28 mice because of either inadequate or no bone marrow sample.

**Figure 1:**
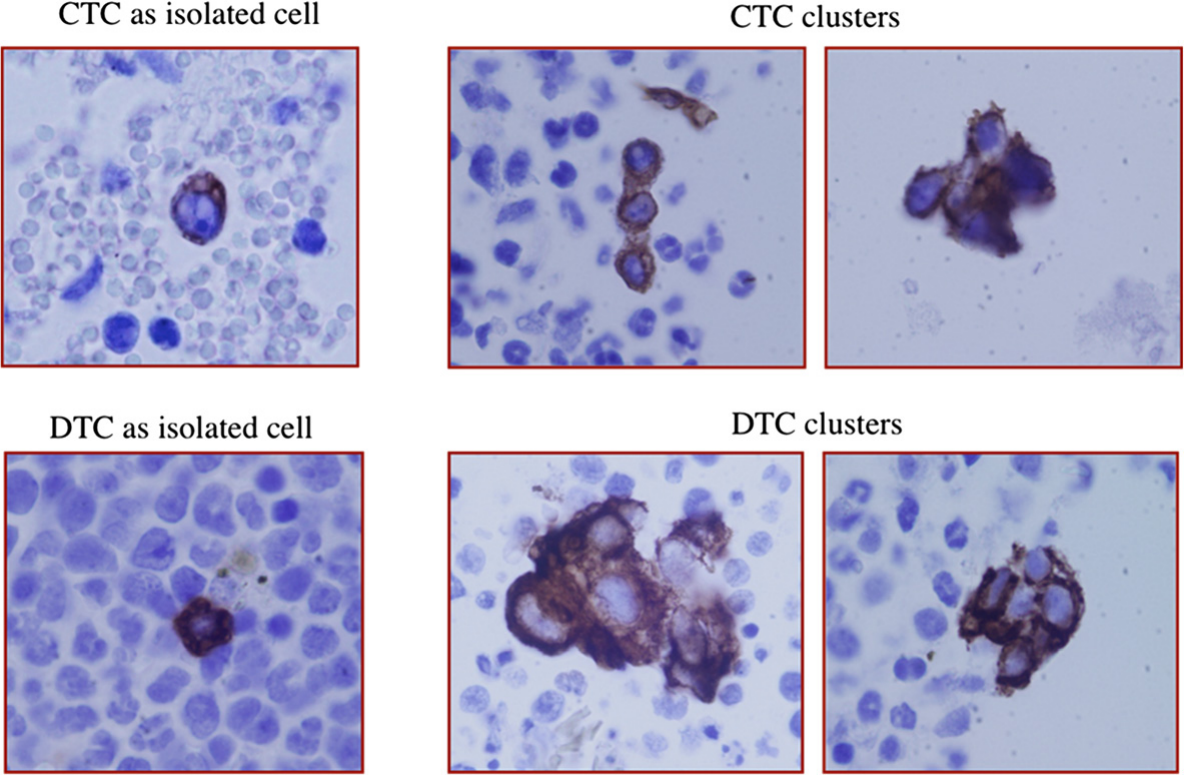
**Representative images of CTCs and BM-DTCs.** Representative IHC images of pan-cytokeratin–positive CTCs and BM-DTCs isolated from mouse peripheral blood and BM, respectively. Left: Representative images of a CTC (upper panel) and a BM-DTC (lower panel) detected as isolated (single) cells. Right: Representative images of CTC clusters (upper panel) and BM-DTC clusters (lower panel).

Among the 18 PDX lines screened, we detected CTCs in 15 (83%) (Table 1). The rate of CTC detection, defined as number of mice positive for CTCs divided by total number of mice tested, for CTC-positive PDX lines ranged from 17% to 100%. CTCs were identified either as individual cells or as clusters of cells that were pan-cytokeratin positive (Figure 1). We found CTC clusters in six of the 18 PDX lines (33%) (Table 1); of these, four were triple-negative (BCM-3204, BCM-3887, BCM-5471, BCM-6257), one was ER/PR/HER2-positive (BCM-4888), and one was ER/PR-positive and HER2-negative (BCM-5097). The maximum number of CTCs detected in a single mouse was 92 per 20 μl of blood, whereas a maximum of 20 to 25 cells was found within a CTC cluster.

BM-DTCs were found in 10 of the 16 PDX lines examined (62.5%). The rate of BM-DTC detection ranged from 20% to 100% in the BM-DTC-positive lines (Table 1). Similar to CTCs, BM-DTCs were identified as individual cells or as clusters (Figure 1). We found that four of 16 (25%) lines had the presence of BM-DTC clusters (Table 1). Two lines (BCM-5471 and BCM-6257) had both CTC and BM-DTC clusters. The highest number of BM-DTCs detected in the bone marrow of a single mouse was 132 per 2 million cells.

LMs, as previously reported [26], were detected in 50% (nine of 18) of PDX lines (Table 1); the detection rate in the LM-positive lines ranged from 6% to 67%.

### Correlation between the presence of CTCs and BM-DTCs and LM

In total, 81 mice were screened in this study (see Additional file 1: Table S1). Of these, 46 mice had both CTCs and BM-DTCs evaluated. The presence of CTCs was strongly associated with the presence of BM-DTCs (*P* = 0.0047, Fisher’s Exact test, Table 2). Even when the data were adjusted for PDX line by using the Cochran-Mantel-Haenszel test, the association was still significant (*P* = 0.0364).

**Table 2:** **Correlation between the presence of CTCs and BM-DTCs in BC PDX-bearing mice**

	**Number of mice with BM-DTCs**	**Number of mice without BM-DTCs**
Number of mice with CTCs	16	13
Number of mice without CTCs	2	15

The data are presented in the 2 × 2 collapsed table, ignoring the strata representing different PDX lines. *P* = 0.0047; Fisher’s Exact test and *P* = 0.0364; Cochran-Mantel-Haenszel test, adjusting for PDX line.

BC, breast cancer; BM-DTC, disseminated tumor cells in bone marrow; CTC, circulating tumor cell; PDX, patient-derived xenograft.

The LM detection rates in these PDX lines were available from the previously conducted studies and hence were evaluated per PDX line rather than per individual mouse [26]. Remarkably, of the nine PDX lines positive for LM, all had detectable CTCs. Conversely, CTCs were detected in only six of the nine lines without LM. Overall, among the 13 PDX lines that had detectable CTCs and had both BM-DTCs and LM rates available, all the lines had either BM-DTCs or LM or both (Table 1). In turn, of the 14 lines with BM-DTCs and/or LM, only one did not have detectable CTCs. Despite this high concordance, the presence of CTCs in the 18 PDX lines we screened did not significantly correlate with the occurrence of LM previously reported in the same lines [26] (*P* = 0.46, Fisher’s Exact test). Interestingly however, the detection of CTC clusters was highly associated with lung metastatic potential (*P* = 0.009, Fisher’s Exact test, Table 3). All six lines in which CTC clusters were found also had developed LM (Table 1). When the analysis was restricted to the 13 lines with triple negative BC PDX, this association was also significant (*P* = 0.007, Fisher’s Exact test).

**Table 3:** **Correlation between the presence of CTC clusters and LM in BC PDX-bearing mice**

	**Number of lines with LM**	**Number of lines without LM**
Number of lines with CTC clusters	6	0
Number of lines without CTC clusters	3	9

*P* = 0.009; Fisher’s Exact test.

BC, breast cancer; CTC, circulating tumor cell; LM, lung metastases; PDX, patient-derived xenograft.

### Genetic signature of primary tumor associated with CTCs clusters and LM

**Figure 2:**
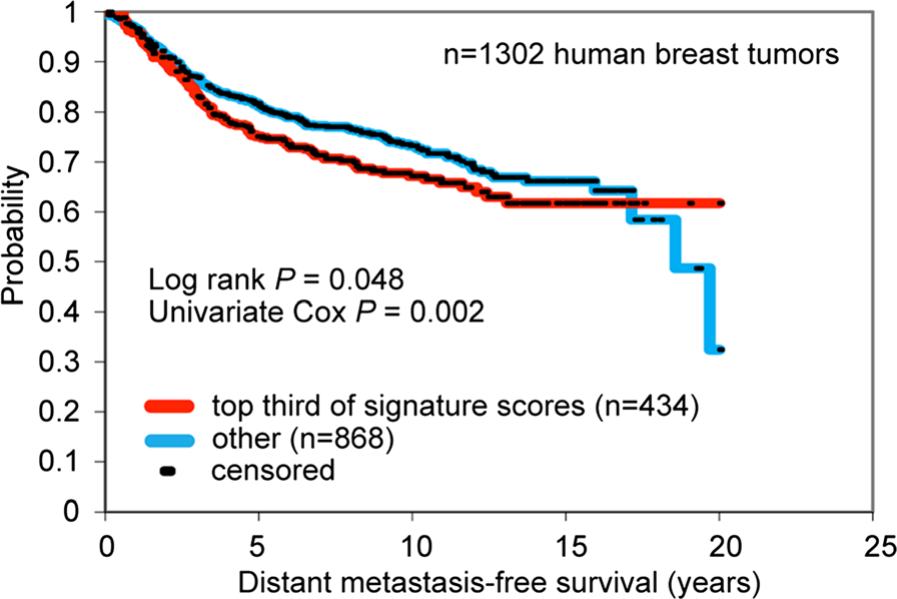
**Prognostic value of the four-gene profile of BC PDXs associated with CTC clusters and LM.** Four genes were found to overlap between two genetic signatures of primary tumor associated with CTC clusters and with lung metastases (LMs). Gene-expression profiles of human BCs were each scored for this signature (the score representing the values of the “high” genes minus the values of the “low” genes). Kaplan-Meier curves compare distant metastasis-free survival in BC patients with relatively higher signature scoring versus those with lower scoring. Univariate Cox evaluates the gene-signature score as a continuous variable. Patient data were extracted from publicly available datasets [28].

Because of the association between the presence of CTC clusters and LM, we wanted to understand the genetic signatures of the primary PDX tumors that give rise to CTC clusters and LM. We computed two separate genetic signatures associated with the presence of CTC clusters and with lung metastatic potential by using the gene-expression arrays of primary tumors from different PDX lines. We identified a set of 35 genes, which formed the CTC cluster-associated signature (see Additional file 2: Table S2). The LM-associated gene signature included 34 genes (Additional file 2: Table S3). Overlapping these two gene signatures resulted in a four-gene profile (Table 4), which was associated with a modest but significant reduction in distant metastases-free survival in a large compendium of publicly available datasets of early BC patients (log-rank *P* = 0.048, 10-year survival probability of 67% for patients in the top 33% of signature scores versus 73% for the rest of the patients) (Figure 2). This gene profile included two genes (*HLA-DP1A* and *GJA1*) that were downregulated and two genes (*PEG3* and *XIST*) that were upregulated (Table 4).

**Table 4:** **Gene profile of BC PDX primary tumors associated with CTC clusters and LM**

**Genes**	**Average fold change in lines with CTC clusters versus no CTC clusters**	**Average fold change in lines with LM versus no LM**
HLA-DP1A*	0.05	0.02
GJA1*	0.04	0.03
**PEG3^**	42.42	25.63
**XIST^**	17.03	43.5

*Downregulated genes; ^Upregulated genes.

BC, breast cancer; CTC, circulating tumor cell; LM, lung metastasis; PDX, patient-derived xenograft.

## Discussion

Multiple lines of evidence suggest that CTCs and BM-DTCs can be used to study the metastatic process and be evaluated in “real-time” to monitor the molecular changes in progressing tumors. However, their use has been largely limited because of challenges in their isolation as well as the very low yield of cells detected in human subjects, especially in the early stages of disease. As an alternative strategy, we established the conditions for the detection of human CTCs and BM-DTCs in unique BC PDX models in this study. As previously shown, these preclinical models accurately resemble their parental human tumors in their molecular features and biologic behavior [26]. Important in this study, we have found that BC PDX models can provide a continuous and renewable source of human CTCs and BM-DTCs. In support of our findings in BC, a recently published study showed that CTCs isolated from pancreatic adenocarcinoma PDX-bearing mice also represent a reliable tool to predict and monitor treatment response [29].

The rate of CTC and DTC detection (83% and 62.5%, respectively) in our studies with PDX models is higher than that reported in the literature for non-metastatic breast cancer patients. We attribute this to the differences in the CTC/DTC detection methods as well as evaluation of larger blood and bone marrow volume relative to the body size of PDX mice than that of patients, as elaborated here. First, most of the commercial techniques to detect CTCs have relied on the presence of a limited number of epithelial markers (that is, epithelial cell adhesion molecule (EpCAM) and/or “epithelial” cytokeratins 8, 18, 19) [30]. This approach likely omits CTCs with a predominant mesenchymal phenotype and a lack of the epithelial markers [25,31-33]. In our approach for CTC/DTC detection, we used a quantitative immunohistochemistry assay to examine “human” tumor cells for the expression of multiple cytokeratin subtypes. Indeed, the pan-cytokeratin antibodies (AE1/AE3) we used bind to multiple cytokeratins present on both human epithelial and mesenchymal cells [34,35]. Second, the higher CTC/DTC detection rate may also result from accessibility to large amounts of peripheral blood and bone marrow, relative to the small size of the mouse body. This is in contrast with only small blood and bone marrow volume used to assess CTC and DTCs in patients. These factors, as well as the immunodeficiency status of the PDX models, may contribute to the higher CTC/DTC detection rates we find in PDX models. In future, it will be of interest to compare CTCs/DTCs from patients side-by-side with those derived from matched PDXs as well as to compare characteristics of CTCs/DTCs within various immunodeficiency models and versus those with reconstituted immune components [36].

The observation of clusters of CTCs and BM-DTCs in the BC PDX-bearing mice is of great interest and further justifies the use of our PDX lines as clinically representative models to study these cells. Several studies have identified multicellular CTC clusters in BC [37,38] and other types of cancer patients [39-43]. In a recent study, CTC clusters were found in 26% of patients with small cell lung cancer, and their presence was an independent prognostic factor [39]. Moreover, CTC clusters isolated from BC patients had high expression of mesenchymal markers and relatively low expression of epithelial markers, suggesting a potential link between the generation of CTC clusters and the EMT process [33]. However, the role of CTC clusters in cancer metastatic dissemination remains unclear. In our study, we found a significant association between the presence of CTC clusters and lung metastatic potential. Only one other study, to our knowledge, has shown a similar association in patients with clear cell renal cell carcinoma [41]. The infrequent finding of clusters of CTCs and BM-DTCs in other studies may be related to the isolation and detection techniques. It is possible that our method of processing the blood and bone marrow fractions may facilitate the detection of clusters.

Of note, we found variability in the detection of CTCs and BM-DTCs in different mice within the same PDX line. The same variability was also present in the previously reported LM detection among these lines [26]. This variability may be attributed to the intratumoral heterogeneity that is commonly seen in patients or some host-specific factors that may influence tumor initiation and progression. However, this observation emphasizes that the future studies to understand the influence of CTCs in distant metastases should include the analysis at a mouse level as well as an overall analysis for the PDX line. In addition, our finding of a significant correlation between the presence of CTCs and BM-DTCs within the same mice is consistent with what is observed in early BC patients [17]. All the BC PDX lines that had CTCs also had BM-DTCs and/or LM. This high concordance rate suggests that these CTCs and BM-DTCs are early indicators of metastatic potential and as such are important for molecular characterization and tumor biology studies. Because BM-DTCs were not evaluated in sufficient numbers of mice for most lines, correlation analyses as well as gene-expression analyses in the PDX lines were restricted to only the CTCs and LM data in this study. Future studies to characterize molecular profiles of CTCs, BM-DTCs, and lung lesions may uncover important biomarkers and treatment targets to prevent metastases.

The four-gene profile we obtained by overlapping the two genetic signatures of primary PDX tumors associated with CTC clusters and with LM was associated with a significant reduction in distant metastases-free survival in early BC patients. However, the observed association was weak because of limitations such as sample size and confounding variables, such as different subtypes and various treatments. Future studies using additional PDX lines are also necessary to validate this gene profile. In support of the derived gene profile, however, other reports have independently identified some of these genes to be associated with LM in BC. For example, *HLA-DP1A*, which encodes a transmembrane protein involved in the antigen-presentation process, was downregulated in both the CTC clusters and LM signatures. Interestingly, *HLA-DP1A* was one of the genes downregulated in the LM signatures generated in two independent studies in BC [44]. The other downregulated gene in our four-gene set was *GJA1*, which encodes a major protein in gap junctions. *GJA1,* also known as connexin *43 (*Cx43), has been shown to suppress mammary tumor metastasis to the lung in a mouse model [45]. Specifically, missense mutation (G60S) in this gene led to production of an altered Cx43 protein that acts in a dominant-negative fashion to disrupt gap-junction assembly and function. This mutation was associated with higher rates of LM in ErbB2-overexpressing mice. Of the two upregulated genes in our four-gene profile, XIST is a noncoding RNA gene on X chromosome and plays a major role in X inactivation. Although overexpression of XIST has been seen in BRCA-1-associated BCs, which typically metastasizes to lungs [46,47], a direct link between XIST and LM in BC has not been established. The other upregulated gene, *PEG3*, encodes a C2H2 type zinc finger protein implicated in regulation of body temperature, feeding behavior, and obesity [48], as well as growth, apoptosis, and maternal nurturing behavior [49]. The role of PEG3 in BC and LM is not clear and warrants further investigation.

## Conclusion

The analysis of CTCs and BM-DTCs in the clinical setting is challenging and imposes multiple limitations. In this study, we provide the first evidence that BC PDX models represent a novel and promising experimental resource for investigating the role of CTCs and BM-DTCs in promoting overt metastases in BC and for their characterization to identify new treatment targets.

## Additional files

Additional file 1: Table S1. Individual mouse data for CTCs and BM-DTCs for all PDX lines screened. Additional file 2: Tables S2 and S3. S2. Gene signature of BC PDX primary tumors associated with the presence of CTC clusters. S3. Gene signature of BC PDX primary tumors associated with the presence of lung metastases.

## Abbreviations

BC: Breast cancer
BM-DTC: bone marrow disseminated tumor cells
CTC: circulating tumor cells
Cx43: Connexin 43
EDTA: ethylenediaminetetraacetic acid
EMT: epithelial-to-mesenchymal transition
EpCAM: epithelial cell adhesion molecule
ER: estrogen receptor
FDR: false discovery rate
HER2: human epidermal growth factor receptor 2
IHC: immunohistochemistry
LIMMA: LInear Models for MicroArray data
LM: lung metastases
PBS: phosphate-buffered saline
PDX: patient-derived xenograft
PR: progesterone receptor
RBC: red blood cell
