# Supplementary material for: Circulating and disseminated tumor cells from breast cancer patient-derived xenograft-bearing mice as a novel model to study metastasis
Source: Breast Cancer Res. 2015 Jan 9;17(1):3. doi: 10.1186/s13058-014-0508-5 (PMC4318479; doi:10.1186/s13058-014-0508-5)
Supplement: Additional file 2: Tables S2 and S3. — S2. Gene signature of BC PDX primary tumors associated with the presence of CTC clusters. S3. Gene signature of BC PDX primary tumors associated with the presence of lung metastases. [file 13058_2014_508_MOESM2_ESM.docx]

**Additional file 2: Table S2.** Gene signature of BC PDX primary tumors consisting of 35 genes associated with the presence of CTC clusters.

**Table S3.** Gene signature of BC PDX primary tumors consisting of 34 genes associated with the presence of lung metastases.
